# Synthetic feedback control using an RNAi-based gene-regulatory device

**DOI:** 10.1186/s13036-015-0002-3

**Published:** 2015-04-14

**Authors:** Ryan J Bloom, Sally M Winkler, Christina D Smolke

**Affiliations:** Department of Bioengineering, Stanford University, 443 Via Ortega, MC 4245, Stanford, CA 94305 USA

**Keywords:** MicroRNA, Ribozyme, Feedback control, Synthetic biology

## Abstract

**Background:**

Homeostasis within mammalian cells is achieved through complex molecular networks that can respond to changes within the cell or the environment and regulate the expression of the appropriate genes in response. The development of biological components that can respond to changes in the cellular environment and interface with endogenous molecules would enable more sophisticated genetic circuits and greatly advance our cellular engineering capabilities.

**Results:**

Here we describe a platform that combines a ligand-responsive ribozyme switch and synthetic miRNA regulators to create an OFF genetic control device based on RNA interference (RNAi). We developed a mathematical model to highlight important design parameters in programming the quantitative performance of RNAi-based OFF control devices. By modifying the ribozyme switch integrated into the system, we demonstrated RNAi-based OFF control devices that respond to small molecule and protein ligands, including the oncogenic protein E2F1. We utilized the OFF control device platform to build a negative feedback control system that acts as a proportional controller and maintains target intracellular protein levels in response to increases in transcription rate.

**Conclusions:**

Our work describes a novel genetic device that increases the level of silencing from a miRNA in the presence of a ligand of interest, effectively creating an RNAi-based OFF control device. The OFF switch platform has the flexibility to be used to respond to both small molecule and protein ligands. Finally, the RNAi-based OFF switch can be used to implement a negative feedback control system, which maintains target protein levels around a set point level. The described RNAi-based OFF control device presents a powerful tool that will enable researchers to engineer homeostasis in mammalian cells.

**Electronic supplementary material:**

The online version of this article (doi:10.1186/s13036-015-0002-3) contains supplementary material, which is available to authorized users.

## Background

The complexity of mammalian cells is largely due to the sophisticated control mechanisms employed to modulate the expression of endogenous genes [1]. Molecules in the cell are organized into networks, which can respond to changes in intracellular protein or metabolite levels, as well as changes in the environment, by upregulating or downregulating the expression of any number of genes [1]. These genetic circuits are essential for virtually all cellular processes including differentiation [2], replication [3], and signal transduction [4]. The ability to interface with these endogenous programs to create novel molecular networks would greatly advance our cellular engineering capabilities for a variety of applications. Central to our ability to create these new genetic circuits is the development of biological components that can respond to changes in the cellular environment and interface with endogenous molecules to enable more sophisticated synthetic circuits [5].

MicroRNAs (miRNA) are a class of small regulatory RNAs that are used to post-transcriptionally control gene expression in higher eukaryotes [6]. MiRNA biogenesis begins with longer pri-miRNAs, which are cleaved by a Microprocessor complex into shorter ~60 nucleotide pre-miRNA hairpins and subsequently exported from the nucleus. The pre-miRNA is subject to a final cleavage step in the cytoplasm by the enzyme Dicer, which creates a 22–25 nucleotide (nt) double stranded RNA [7]. One of these RNA strands, called the guide strand, is then incorporated into one of the Argonaut family of proteins to create the RNA-induced silencing complex (RISC). This complex subsequently cleaves or translationally represses the target transcript depending on the degree of complementarity between the miRNA and the target [6]. This powerful mechanism of gene regulation plays vital roles in diverse processes, such as development and angiogenesis, and it is estimated that RNAi regulates the majority of genes in the human genome [8].

RNA-based synthetic biology tools are powerful resources for building synthetic control systems that can detect and respond to changes in cell state or the cellular environment, and synthetic RNA-based systems have been demonstrated in mammalian cells [9-12]. Frameworks have been described for the design of miRNA- and shRNA-based molecular switches, whose biogenesis is responsive to changes in concentration of intracellular ligand molecules [13]. In general, these regulators are constructed by coupling the binding of a ligand to an RNA aptamer with the proper formation and biogenesis of the RNAi element [13]. The physical coupling of the aptamer to the RNAi element has been accomplished in several ways. Researchers have directly coupled an aptamer to a pri-miRNA, such that binding of the ligand to the aptamer sterically inhibits proper biogenesis by the Microprocessor [13]. Both direct coupling and strand displacement mechanisms have been described, where ligand binding to the aptamer changes the structure of an shRNA such that it is unable to be properly processed by Dicer [14,15].

These RNAi-based control systems are particularly useful for imparting conditional silencing over endogenous genes. Previously, we developed a computational model that predicts target gene expression levels resulting from synthetic circuits based on miRNA regulatory components [16]. This model was applied to direct the rational design of miRNA-based genetic circuits and supported an *in-vivo* protein concentration detector circuit by providing a quantitative relationship between target reporter and protein ligand levels in the cell. However, the designs in this previous work utilize a switch architecture that inhibits RNAi-based gene silencing in the presence of the ligand, thereby increasing target gene expression as a function of increasing ligand concentration and operating as ON switches. An RNAi-based control platform that can be used to decrease the expression of a target gene in response to increasing concentrations of proteins of interest has not been described. However, this type of OFF switch regulation is essential for building core control strategies such as negative feedback control. Negative feedback is a prevalent control mechanism found in diverse biological systems, such as bacterial chemotaxis [17] and vision [18], that can be utilized to achieve homeostasis.

Here we describe and characterize an OFF genetic control device based on RNAi that combines a ligand-responsive ribozyme switch and synthetic miRNA regulators. The device architecture links ribozyme cleavage to miRNA levels, such that ligand binding modulates ribozyme cleavage rates and thus miRNA-based gene silencing. The system was prototyped using a previously described theophylline-responsive ribozyme switch and a miRNA that targets a fluorescent reporter. A mathematical model of this system was developed to highlight important design parameters in programming the quantitative performance of RNAi-based OFF control devices. We demonstrated RNAi-based OFF control devices that respond to protein ligands by incorporating protein-responsive ribozyme switches to E2F1 and MS2. We utilized the OFF control device platform to build a negative feedback control system that maintains target intracellular protein levels around a set point. The negative feedback control system acts as a proportional controller, maintaining target intracellular protein levels in response to increases in transcription rate. The described RNAi-based OFF control device presents a powerful tool that will enable researchers to engineer homeostasis into mammalian cells, for example, to maintain a desired phenotype even in the presence of genetic mutations or fluctuating levels of signaling molecules or cytokines.

## Results and discussion

### A trans-acting genetic device that exhibits OFF control by coupling ribozyme switches and miRNAs

**Figure 1:**
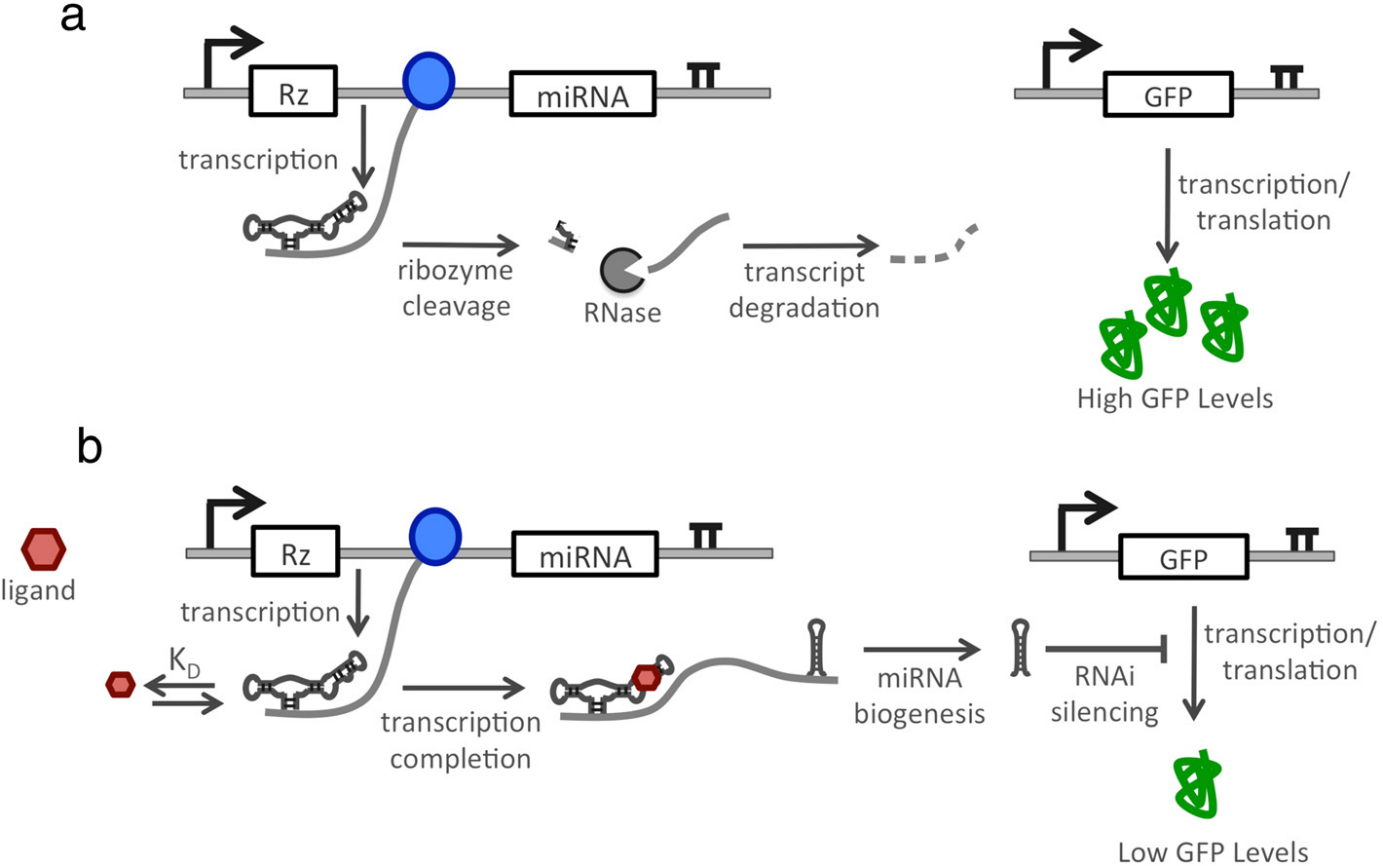
Schematic of the RNAi-based OFF control device architecture. A catalytic ribozyme switch is placed upstream of a pri-miRNA in the same expression cassette. **(a)** In the absence of the input ligand, the ribozyme switch cleaves once it is transcribed, aborting transcription such that the miRNA is not be transcribed, thereby resulting in high target gene expression levels. **(b)** In the presence of ligand, ribozyme cleavage is inhibited such that transcription proceeds through the pri-miRNA, which undergoes proper miRNA biogenesis. Target expression levels are lowered due to miRNA silencing.

We developed a strategy to implement a gene-regulatory device that exhibits OFF control in the regulation of endogenous gene targets. Our platform design is inspired by a mechanism found in natural miRNA clusters, where cleavage of a transcript in a region upstream of a pri-miRNA reduces the steady-state level of the transcript encoding the pri-miRNA and thus the resulting gene silencing due to the processed miRNA [19]. We hypothesized that by imparting conditional cleavage of a transcript in a region upstream of a pri-miRNA by using a ligand-responsive ribozyme we could control the level of silencing from the encoded miRNA (Figure 1). In the proposed system design, the ribozyme cleaves in the absence of ligand, thereby reducing the steady-state level of the pri-miRNA transcript and resulting in reduced miRNA-based silencing and increased target gene expression. However, when bound to the ligand the ribozyme’s cleavage rate is substantially reduced, such that higher steady-state levels of the pri-miRNA transcript will accumulate and be processed, resulting in reduced expression of the target gene.

We used a modular ligand-responsive ribozyme switch platform that supports efficient tailoring of RNA device function [20]. The ribozyme switch platform specifies physical linkages between three functional RNA components: a sensor encoded by an RNA aptamer, an actuator encoded by a hammerhead ribozyme, and a transmitter encoded by a sequence that undergoes a strand-displacement event (Additional file 1: Figure S1). The modular nature of this ribozyme design makes it relatively straightforward to modify the ligand-responsiveness of the switch by swapping in new aptamer sequences into the sensor component of the platform while maintaining the overall activity of the switch.

**Figure 2:**
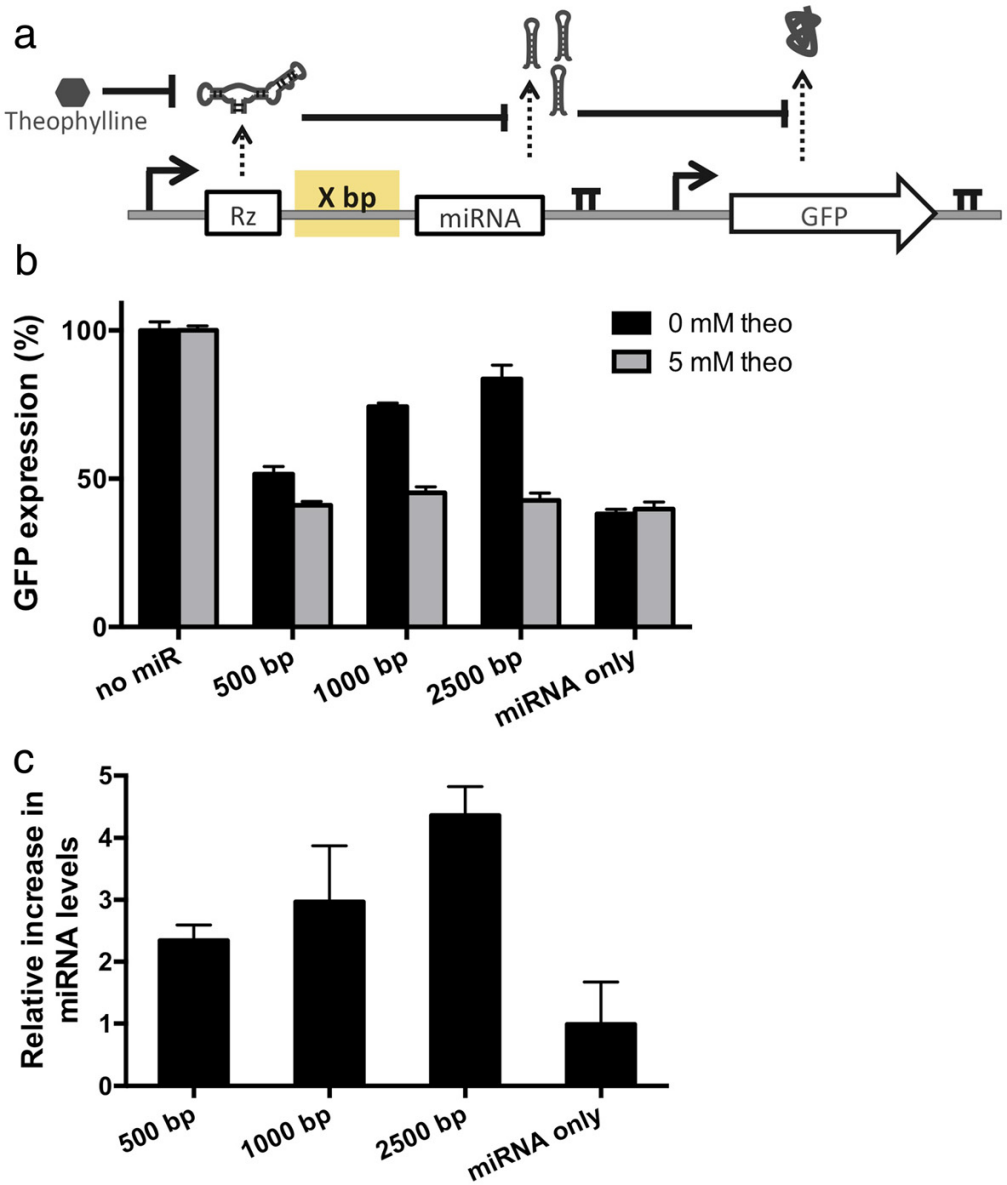
The effect of different spacer lengths on performance of the RNAi-based OFF control device. **(a)** Schematic of the expression cassettes used to test the OFF control device architecture. A theophylline-responsive ribozyme switch was placed at various distances upstream of a miRNA targeting GFP. GFP was expressed constitutively under a separate expression cassette. Both constructs were stably integrated into an HEK293 Flp-In cell line. **(b)** GFP expression in the absence and presence of theophylline from OFF control devices with varying spacer lengths. GFP levels were measured 48 hours after the addition of 0 or 5 mM theophylline. No miR and miRNA only represent constructs with a theophylline-responsive ribozyme switch and no downstream miRNAs (negative control) and a construct with miRNA only upstream of a GFP-targeting miRNA (positive control), respectively. Data is reported as the percent of GFP expression in a cell line with no miRNA present. Error bars represent ±1 standard deviation of at least three independent experiments. **(c)** Relative increase in miRNA levels after addition of theophylline. Relative miRNA levels are reported 48 hours after the addition of 0 or 5 mM theophylline and normalized to the miRNA levels measured from identical cell lines in the presence of theophylline. Error bars represent ±1 standard deviation of three independent experiments.

An expression cassette for the device was designed based on placing a ligand-responsive ribozyme upstream of a cluster of three identical GFP-targeting miRNAs (Figure 2a). The miRNA guide strands were designed to be completely complementary to the target transcript, such that their mechanism of gene silencing reflects that of an siRNA although they are processed from a primary transcript through the endogenous miRNA pathway [13]. The device was prototyped with a previously characterized theophylline-responsive ribozyme that was shown to exhibit a reduced cleavage rate as a function of increasing theophylline concentration [21]. Three versions of the OFF control device were designed in which the spacer length between the ribozyme and miRNA components was varied (500, 1500, 2500 bp) to examine the effect of spacer length on device performance. Control constructs were developed that had the theophylline-responsive ribozyme replaced with a non-cleaving ribozyme or that removed the miRNAs from the expression cassette (Additional file 1: Figure S2). A second expression cassette was constructed that encoded the expression of a GFP fluorescent reporter protein. Isogenic cell lines stably expressing both expression cassettes were generated from a Flp-In HEK293 cell line by co-transfecting a plasmid harboring both expression cassettes with a plasmid that expresses a Flp recombinase (pOG44) and selecting for stable integrants.

Device performance was quantified by measuring target protein (GFP) and mature miRNA levels in the presence and absence of ligand (Figure 2b, c). GFP levels were measured using a flow cytometry assay and mature miRNA levels were measured using a qRT-PCR assay with custom TaqMan small RNA probes. The data demonstrate that the longest spacer tested (2500 bp) resulted in the greatest change in target gene expression levels in the presence and absence of ligand (Figure 2b). qRT-PCR analysis showed that the decrease in target gene expression levels correlated with an increase in mature miRNA levels, indicating that the observed changes in target gene expression levels were due to miRNA silencing (Figure 2c). The OFF state (i.e., expression level in the presence of theophylline) measured for all constructs is similar to that from the inactive ribozyme control construct, which sets the expected lower limit for the system (Figure 2b). The ON state (i.e., expression level in the absence of theophylline) generally increased with spacer length, consistent with previous findings in the literature [19]. Overall, the device with the longest spacer length tested (2500 bp) exhibited ON and OFF states very close to the controls (Figure 2b, Additional file 1: Figure S2), indicating that this device was accessing nearly the full potential dynamic range of the system. The data indicate that longer spacer lengths may increase the time between transcription of the ribozyme component and transcription of the downstream miRNA component, thus increasing the probability that the ribozyme will cleave and abort transcription [19] prior to the transcription and processing of the miRNA.

### A computational model informs the design of the RNAi-based OFF control device

We developed a mathematical model to inform the design of the RNAi-based OFF control device to elucidate important system design parameters. The model describes the kinetics of ribozyme cleavage and miRNA formation using a series of differential equations describing the separate processes (Additional file 1: Figure S3, Additional file 1: Text S1). The transcription of the ribozyme-miRNA transcript is modeled as a two-step process in which an “immature transcript”, harboring only the ribozyme component, is initially formed. This immature transcript is either cleaved and subsequently degraded or elongated into a full-length transcript, encoding both the ribozyme and miRNA components. Mature miRNAs are formed from the full-length transcript and subsequently silence target gene expression through enzymatic degradation using a term that resembles traditional Michaelis-Menten kinetics, which includes a constant (*K*_*m*_) describing the affinity between the miRNA and the target transcript [22]. The presence of the input ligand will lower the ribozyme cleavage rate, resulting in an increase in the level of full-length transcript and thus increased miRNA levels. The binding kinetics between the aptamer and the ligand (*k*_*a*_*, k*_*d*_) as well as the cleavage rates of the ribozyme when bound and unbound to the input ligand (*k*_*cleave-bound*_*, k*_*cleave*_) were measured experimentally [23,24]. The parameters that determine the gene silencing at different miRNA levels (*a*, *K*_*m*_) were determined previously [22]. All other model parameters were determined using parameter fitting software in MATLAB by simulating the system of equations and fitting the steady-state values to the experimental data for the theophylline-responsive OFF control device.

**Figure 3:**
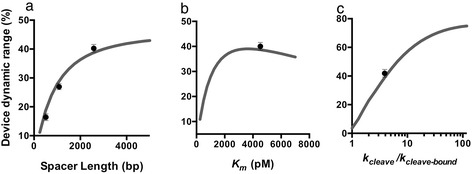
Parameters that affect performance of the RNAi-based OFF control device. **(a-c)** Predicted relationships between device dynamic range and the length of the spacer between the ribozyme switch and miRNA components **(a)**, miRNA-target transcript binding affinity, *K*_*m*_ **(b)**, and the ratio of ribozyme cleavage rates in the absence and presence of ligand, *k*_*cleave*_/*k*_*cleave-bound*_ **(c)**. Grey line: model predictions; circle: experimental results. Device dynamic range is reported as the percent change in GFP expression in the absence and presence of theophylline.

We used the model to predict the effects of different parameters on device function and to identify where the theophylline-responsive device fell in relation to these predictions. For example, the model predicts the quantitative increase in the change in target expression level as a function of increasing spacer length that is observed in our experimental system (Figure 3a). We used the model to interrogate the impact of miRNA-target gene affinity, *K*_*m*_, on the performance of the OFF control device (Figure 3b). The model predicts an optimal value of approximately 3500 nM, corresponding to the largest dynamic range achievable by the OFF control device. At very high *K*_*m*_, there is little target gene silencing from the miRNA, and an increase in miRNA levels will lead to little difference in target gene expression. At very low *K*_*m*_, a large amount of miRNA silencing is observed even at low miRNA levels that saturates as miRNA levels increase [22]. Our experimental results indicate that the *K*_*m*_ of the theophylline-responsive control device is nearly optimal for the system (Figure 3b). Finally, we used the model to interrogate the impact of the ratio of cleavage rates between the ligand unbound and bound ribozyme switch (*k*_*cleave*_*/k*_*cleave-bound*_) on the performance of the OFF control device (Figure 3c). As this ratio increases, the model predicts an increase in the change in target gene expression levels in the presence of the ligand. In our theophylline-responsive system, the ratio of cleavage rates is approximately 5.6. The model predicts that increasing this ratio would lead to substantial increases in the change in target gene expression, indicating that this is a key parameter in the design of control devices with specified dynamic ranges.

The model can be used to identify which components of the circuit can be targeted for modification to optimize the performance of the circuit. For example, both spacer length and the ratio of ribozyme cleavage rates in the presence and absence of the ligand (*k*_*cleave*_*/k*_*cleave-bound*_) were found to be critical parameters in tuning the dynamic range of the OFF control device. High-throughput screening strategies have been described that support efficient tuning of the cleavage rates of ribozyme switches [25]. As another example, the model indicated that miRNA silencing efficiency, largely determined by *K*_*m*_, determines the silencing observed at a given miRNA level. The *K*_*m*_ of a miRNA-target gene pair can be modified by incorporating additional target sites into the 3′ untranslated region (UTR) of the target transcript [22,26]. With the components selected for this study, the systems were limited to dynamic ranges on the order of ~2-fold. While this regulatory activity is on the order of that observed with endogenous miRNA regulatory networks [27], it is lower than many synthetic transcription-based control systems in use. Improvements in the properties of the underlying device components (i.e., ribozyme switch, miRNA) will allow for OFF control devices with improved dynamic ranges supporting broader applications.

### Protein-responsive RNAi-based OFF control devices

To adapt the RNAi-based OFF control devices to respond to proteins, we replaced the small molecule-responsive ribozyme switch with ribozymes that alter their cleavage activity in the presence of specified protein ligands. The modular nature of the ribozyme switch platform allowed us to tailor the ligand responsiveness of the switch by swapping new aptamer sequences into the sensor domain of the platform [20].

We developed protein-responsive ribozyme switches to two protein ligands: the MS2 bacteriophage coat protein (MS2) and an oncogene (E2F1). Specifically, sequences for previously characterized RNA aptamers to MS2 [28] and E2F1 [29] were placed directly into the sensor domain of the ribozyme switch platform using a previously characterized transmitter sequence [25]. The binding and cleavage activities of the MS2- and E2F1-responsive ribozyme switches were characterized using previously described *in vitro* assays. The binding activities of the switches were measured using a surface plasmon resonance assay on a Biacore instrument [23]. The results of this binding assay indicate that the protein-responsive switches bind their cognate ligands with high affinities; the MS2-responsive switch has a K_D_ of 296 nM [30] and the E2F1-responsive switch has a K_D_ of 548 nM (Additional file 1: Figure S4a). The cleavage rate of the MS2-responsive switch was measured using a gel-based cleavage assay [23]. The results of the cleavage assay demonstrate that the MS2 ligand substantially affected the cleavage rate of the ribozyme switch, resulting in an approximate 2.5-fold decrease in cleavage rate in the presence of 2 μM MS2 [30]. End point cleavage assays were similarly performed on the E2F1-responsive switch and confirmed that the presence of ligand decreased the cleavage activity of this ribozyme switch (Additional file 1: Figure S4b, c). Taken together, the data indicate that the protein-responsive ribozyme switches bind their cognate ligands with high affinities and in a manner that disrupts cleavage activity of the ribozyme.

**Figure 4:**
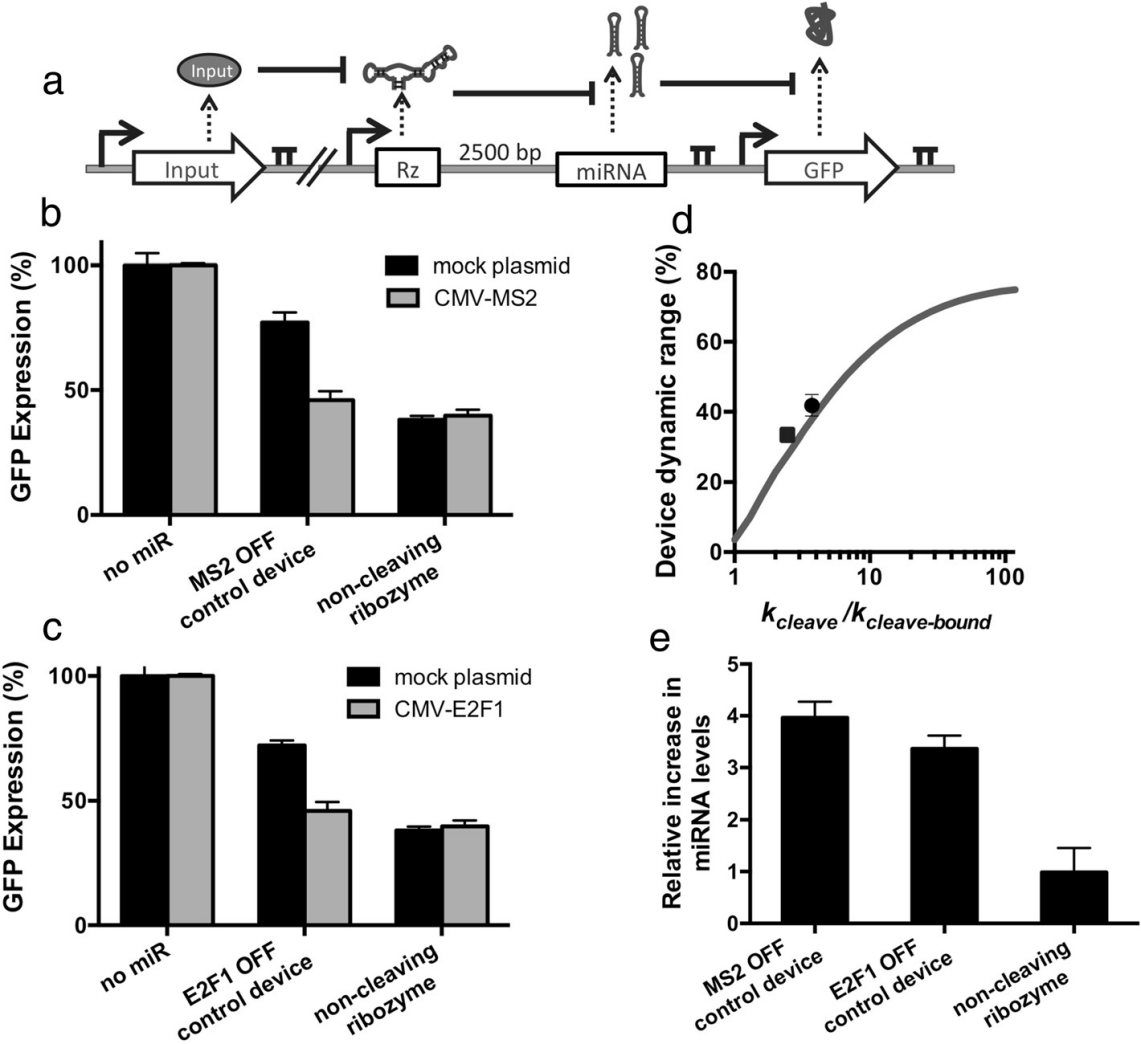
Protein-responsive RNAi-based OFF control devices. **(a)** Schematic overview of the genetic circuit used to characterize protein-responsive OFF control devices. The OFF control devices and GFP were stably integrated and expressed from identical expression cassettes as described in Figure 2a, except that the theophylline-responsive ribozyme was replaced with an MS2- or E2F1-responsive ribozyme switch. A plasmid encoding the expression of DsRed only (mock plasmid) or the protein ligand (MS2 or E2F1) was transfected into the cell lines to measure activity of the OFF control device. **(b-c)** GFP expression levels under the control of the MS2-responsive **(b)** or E2F1-responsive **(c)** OFF control device. No miR and miRNA only represent constructs with a protein-responsive ribozyme switch and no downstream miRNAs (negative control) and a construct with a non-cleaving ribozyme upstream of a GFP-targeting miRNA (positive control), respectively. Error bars represent ±1 standard deviation of at least three independent experiments. **(d)** Device dynamic ranges for the OFF control devices as a function of the ratio of cleavage rates (*k*_*cleave*_/*k*_*cleave-bound*_). Grey line: model; circle: theophylline-responsive device; square: MS2-responsive device. Device dynamic range is reported as the percent change in GFP expression in the absence and presence of theophylline. **(e)** Relative increase in miRNA levels after addition of protein ligand. Relative miRNA levels are reported normalized to the miRNA levels measured from identical cell lines in the absence of protein ligand. Error bars represent ±1 standard deviation of three independent experiments.

The validated protein-responsive ribozyme switches were placed into the OFF control device platform to build an RNAi-based control system that decreased target gene expression levels as a function of increasing MS2 and E2F1 levels. Expression cassettes were constructed with the protein-responsive ribozymes placed 2500 bp upstream of a GFP-targeting miRNA (Figure 4a)*.* A second expression cassette was constructed that encoded the expression of a GFP fluorescent reporter protein. Isogenic cell lines stably expressing both expression cassettes were generated from a Flp-In HEK293 cell line as previously described.

Device performance was quantified by measuring target protein (GFP) and mature miRNA levels in the presence and absence of the protein ligands as previously described (Figure 4b-e). Plasmids expressing MS2 or E2F1 were transiently transfected into the stable cell lines expressing the MS2- or E2F1-responsive OFF control devices, respectively, and compared to the identical stable cell line transfected with a mock plasmid (i.e., no protein ligand expressed). The data demonstrate that GFP expression levels are reduced up to 2.1- and 1.8-fold in response to MS2 and E2F1 expression, respectively (Figure 4b,c). As with the theophylline-responsive OFF control device, the OFF states of the protein-responsive devices are similar to the positive control (i.e., miRNA only). When taking into account the differences in cleavage rates (*k*_*cleave*_*/k*_*cleave-bound*_) between the protein-responsive ribozymes and the theophylline-responsive ribozyme, with no additional refitting of other parameters, the model predicts the observed difference in gene-regulatory activities of the devices (Figure 4d). Larger changes in target gene expression correlated with larger cleavage rate ratios in the presence and absence of ligand, supporting that the cleavage rate ratio is a key design parameter for these systems. The qRT-PCR analysis indicated that the increase in GFP silencing was due to an increase in the levels of mature miRNAs within the cells (Figure 4e). Taken together, these results indicate that OFF control devices can be constructed to respond to a variety of ligands by integrating ribozyme switches that respond to the ligands of interest.

### Protein-responsive RNAi-based OFF control devices support the implementation of negative feedback control systems

Feedback regulation is ubiquitous in biology and fundamental to many engineering applications. Synthetic biologists have designed a wide range of engineered biological transcriptional feedback regulation systems [31,32]. Negative and positive transcriptional feedback controllers have been used to stabilize desired output protein concentrations, accelerate system dynamics, and generate biological oscillations [33-35]. However, engineered genetic negative feedback control systems have been used to regulate the expression of transgenes; a system has not been developed that can exert feedback control over endogenous genes. This expanded capability would be a powerful tool to engineer synthetic circuits to maintain desired cell phenotypes, for example, in the presence of genetic mutations or fluctuating levels of signaling molecules or cytokines.

The RNAi-based OFF control device has the ability to regulate the levels of a target protein around a desired set point by enabling the implementation of a negative feedback control system in mammalian cells (Additional file 1: Text S1). In this system design, a ribozyme switch responsive to a protein of interest and a miRNA that targets that same protein of interest are integrated into the OFF control device architecture. Thus, an increase in levels of the input protein will result in an increase in levels of the miRNA, which will subsequently reduce levels of the protein to the desired set point level.

**Figure 5:**
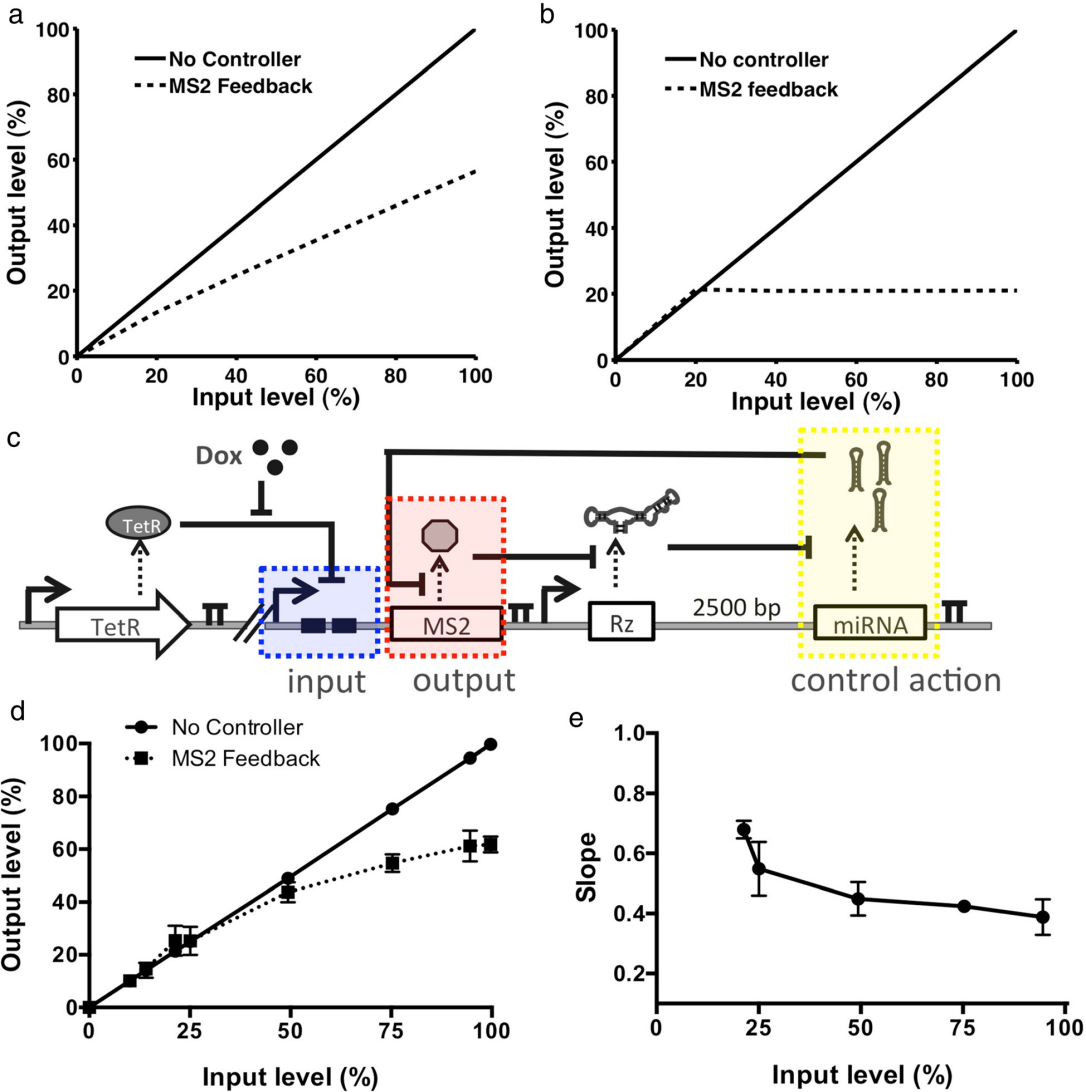
The protein-responsive OFF control device supports the design of a negative feedback control system over MS2 levels. **(a)** Model prediction for MS2 feedback system where miRNA level (control action) is dependent upon the level of miRNA in the cell. The output (MS2 protein) levels are shown plotted as a function of the input (maximum MS2) levels. **(b)** Model prediction for the MS2 system where the miRNA level (control action) is independent of the levels of miRNA. The output (MS2 protein) levels are shown plotted as a function of the input (maximum MS2) levels. **(c)** Schematic of the circuit used to test the MS2-responsive OFF control device exerting feedback control over MS2 levels. A doxycycline-responsive CMV-TetO2 promoter was used to control the expression of an MS2-DsRed fusion protein. An OFF control device containing an MS2-responsive ribozyme switch and a miRNA targeting DsRed were expressed in a separate expression cassette under the control of a constitutive promoter (P_CMV_). The expression cassettes are stably integrated into a Flp-In HEK293 T-REx cell line, which expresses the TetR repressor. Input levels (maximum MS2-DsRed levels) were varied by growing the cells under different concentrations of doxycycline. The input is highlighted in red, the output (MS2-DsRed levels) is highlighted in blue, and the control action (miRNA levels) is highlighted in yellow. **(d)** Output (MS2-DsRed) levels as a function of the input (maximum MS2-DsRed transcript) levels for circuits with and without a feedback controller. The output and input are reported as the percent of the maximum DsRed levels in a cell line with no miRNA present. Error bars represent the standard deviation of three independent experiments. **(e)** The slope as a function of the input. The local slope is calculated as: Slope_*i*_ = (Output_*i*_ – Output_*i-1*_) /(Input_*i*_ – Input_*i-1*_).

We first adapted our model to capture the behavior of a negative feedback system based on our protein-responsive OFF control device (Additional file 1: Text S1). In the negative feedback system model, the input is the maximum level of target gene expression, the output is the target protein levels, and our control action, or miRNA levels, regulates the ratio of output to input levels such that target protein expression remains relatively consistent independent of input level. In order to capture the behavior of a feedback control system which regulates levels of MS2-DsRed fusion protein, we included the parameters for the cleavage rate of the MS2-responsive ribozyme and a DsRed-targeting miRNA in the model (Additional file 1: Text S1). In this way the output (MS2-DsRed protein levels) directly affects miRNA levels, and therefore at higher MS2-DsRed levels, the increase in miRNA levels will decrease levels of MS2-DsRed protein. The model predicts that in the parameter ranges observed experimentally the OFF control device exerts proportional control (P-control) over the protein of interest. Unlike in an integral controller, the output from P-type controllers is not independent of the input, but rather exhibits an offset from the desired set point. The result is that the level of our protein of interest (output) will increase linearly as the maximum level (input) increases (Figure 5a). Consistent with observations of feedback control in biological systems [18], the model predicts that the offset will decrease as the degradation of the miRNA depends less on the concentration of the miRNA. If the degradation of the miRNA is a constant rate such that it does not rely on the concentration of miRNA, then ‘perfect adaptation’ or integral control will be achieved. Integral control is characterized by no offset and the steady-state output protein levels are stable at a constant level, regardless of the input level (Figure 5b).

We examined the model predictions in our experimental system by modifying the OFF control device to exhibit negative feedback around the levels of the MS2 protein (Figure 5c). An expression cassette was designed in which the MS2-DsRed fusion protein was under the transcriptional control of a doxycycline-inducible CMV-TetO2 promoter and TetR transcriptional repressor pair. In a separate expression cassette, the MS2-responsive OFF control device was modified such that the GFP-targeting miRNA component was replaced with a DsRed-targeting miRNA. In this negative feedback system design, the MS2-DsRed protein levels serve as the system output. As the output levels increase, the MS2 will trigger the formation of miRNAs that target the coding region of DsRed, thereby acting to reduce the levels of MS2-DsRed and serving as a form of negative feedback control. Isogenic cell lines stably expressing both expression cassettes were generated from a Flp-In HEK293 T-REx cell line as previously described. The Flp-In HEK293 T-REx cell line also stably expresses the TetR transcriptional repressor, such that the maximum level of MS2-DsRed (the input) is tightly regulated by the concentration of doxycycline in the media. In particular, the levels of MS2-DsRed were held low prior to induction and measurements were made 48 hours after induction, such that differences in the half lives of d2EGFP and MS2-DsRed (2 hours [36] versus 8 hours [37], respectively) would not affect the experimental results.

The resulting stable cell lines were used to characterize the activity of the negative feedback control device regulating MS2 levels (Figure 5c). DsRed levels were used as the output from the feedback system and were measured using a flow cytometry assay, the input (maximum MS2-DsRed level) was controlled using a doxycycline-responsive inducible promoter. Our data show that, compared to a system with no feedback control, there is a significant reduction in the levels of MS2 protein as the transcription rate is increased (Figure 5d). The local slope was also calculated as: Slope_*i*_ = (Output_*i*_ – Output_*i-1*_) /(Input_*i*_ – Input_*i-1*_) (Figure 5e). Our experimental system (Figure 5d, e) exhibits similar behavior to that predicted of a proportional controller (Figure 5b) in that the output is not independent of the input, but rather linearly increases as the input increases. Our results highlight that the RNAi-based OFF control device platform can be used to effectively exert feedback control over the levels of a protein of interest.

While there are several examples of negative feedback being implemented in synthetic circuits to alter the dynamics of the circuit [34,38], as well as in the construction of oscillators [33] and toggle switches [39], the described control systems do not have the ability to exert feedback control over endogenous genes. Our RNAi-based feedback control system behaves as a proportional controller (P-controller), where the control action (miRNA level) is proportional to the output (protein level). To implement integral feedback control the control action (miRNA level) must be solely a function of the output (protein level). Our model indicates that perfect adaptation cannot be achieved in our system as the miRNA degradation rate is a linear function of the miRNA level. Maintaining miRNA degradation at a constant level would allow us to achieve perfect adaptation [18]. However, in practice, keeping the rate of change of a molecular species independent of its own level through zeroth-order degradation kinetics is difficult to achieve in cellular systems, where molecular concentrations are governed by biochemical kinetics. Any mechanisms working to decrease the concentration of the control action species will likely need to operate at saturation with respect to the control action species in order to achieve zeroth order degradation kinetics. It is likely that many biological feedback systems operate using proportional control and may need to incorporate additional redundancies in cases where maintaining the level of a molecule within a narrow concentration range is critical.

## Conclusions

Our work describes a novel genetic device that increases the level of silencing from a miRNA in the presence of a ligand of interest, effectively creating an RNAi-based OFF control device. Our OFF control device incorporates a ligand-responsive ribozyme and spacer element upstream of a miRNA regulator within the same expression cassette such that co-transcriptional cleavage of the ribozyme aborts transcription and prevents pri-miRNA formation. In the presence of ligand, the cleavage rate of the ribozyme is reduced, resulting in accumulation of pri-miRNA transcripts, which are subsequently processed into mature miRNAs that silence the target gene. Similar to previous designs [40], this coupled architecture allows the miRNA regulator to act as a genetic inverter, by inverting the signal from the ON ribozyme switch to an OFF genetic switch. We demonstrated the flexibility of the OFF switch platform by showing that it can be used to respond to both small molecule and protein ligands and developed a computational model describing the device that revealed important design parameters and parameter ranges associated with optimal performance of the control device. Finally, we used the RNAi-based OFF switch to implement a negative feedback control system, that maintained target intracellular protein levels in response to increases in transcription rate.

Our computational model revealed important design parameters and parameter ranges associated with optimal performance of these control devices. Thus, the model can be used to guide careful selection or tuning of components and/or inform how different parameters would impact the quantitative behavior of the control device. For example, the model identified spacer length, ribozyme cleavage rates in the presence and absence of ligand, and miRNA silencing efficiency as key parameters in the design of OFF RNAi-based control devices.

With the components selected for this study, the systems were limited to dynamic ranges on the order of ~2-fold. While this regulatory activity is on the order of that observed with endogenous miRNA regulatory networks [27], improvements to these activities will increase the broader utility of these devices in synthetic contexts. Future improvements to the dynamic ranges exhibited by OFF RNAi-based control devices can be achieved through refinement of the underlying device components (i.e., ribozyme switch, miRNA).

By leveraging an RNAi-based regulatory mechanism, the described OFF control device can be adapted to exert negative feedback control over endogenous gene targets. The described control platform can be used in future cellular engineering and gene therapy applications, where the expression of key genes can be kept at desired levels even in the presence of cellular mutations or malfunctioning signaling pathways. Further development and optimization of the ribozyme switch and miRNA components will enable researchers to extend this platform for implementation into applications in health and medicine.

## Methods

### Plasmid construction

Modern molecular biology techniques were used to create the plasmids and cell lines used in this work. A chemically-competent *Escherichia coli* strain TOP10 was used for cloning purposes (Life Technologies, Carlsbad, CA) and was grown in LB medium (Becton, Dickinson and Company (BD), Franklin Lakes, NJ) with the indicated antibiotic concentrations. Custom oligonucleotides were synthesized by Integrated DNA Technologies (IDT, Coralville, IA) and the Stanford Protein and Nucleic Acid Facility (PAN, Stanford, CA). PCR was performed with Taq polymerase (New England Biolabs, Ipswich, MA) for products less than 1 kb and Pfu Ultra II Hotstart (Agilent Technologies, Santa Clara, CA) for products greater than 1 kb. All restriction enzymes were obtained from New England Biolabs. Plasmids were prepared from *E. coli* using Econospin columns (Epoch Life Science, Missouri City, TX) according to manufacturer’s instructions. Sequencing was performed by Elim Biopharmaceuticals, Inc (Hayward, CA).

The plasmid pCS2304 was constructed by PCR amplifying a DNA fragment encoding a CMV promoter, d2EGFP coding sequence, the BGH-polyA tail, using the primers BglII CMV Fwd (5′-AATAAGATCTTAGTTATTAATAGTAATCAATTACGGGGTCATTAGT-3′) and Mfe bgh Rev (5′-TATTCAATTGTTTGCTGTTTCGTCCTCCG-3′) and cloning the fragment between the BglII and MfeI restriction sites in pcDNA5/FRT. Spacer regions of different lengths were PCR amplified from the LacZ gene using the primers BstBi-Xma-mid LacZ Fwd: (5′-ATATTCGAAAATACCCGGGA TACTGTCGTCGTCCCCT-3′) and 500 bp LacZ rev (5′-AATAGGTACCATCGATAAT AACCGGTGTGTAGATGGGCGCATCG-3′) for the 500 bp spacer, 1000 bp LacZ rev (5′-AATAGGTACCATCGATAATAACCGGTTTG GCCCTGTCAGAAATCCAGGGG-3′) for the 1000 bp spacer, and 2500 bp LacZ rev (5′-AA TAGGTACCATCGATAATAACCGGTAAGCGCTGTCACCGGAATCAA-3′) for the 2500 bp spacer. These fragments were subsequently digested with BstBI and BamHI and cloned between the BstBI and BamHI restriction sites in the above intermediate plasmid to create pCS2722 (Additional file 1: Figure S5). The Rev primers used for amplifying the spacer incorporated AgeI and ClaI restriction sites on the 3′ end of the spacer, which were used for miRNA cloning in downstream steps. The resulting plasmids include two expression cassettes, one that drives the expression of d2EGFP and one that drives expression of different spacers that include restrictions sites on the 5′ end (BstBI, XmaI) and 3′ end (AgeI, ClaI) and is the base plasmid which was used in all OFF control switch studies in this work. The BstBI and XmaI restriction sites were used to clone the ribozyme switches and associated controls, and the AgeI and ClaI restriction sites were used to clone the miRNAs and associated controls. Finally, the plasmid contains FRT recombinase sites for making stable cell lines with the FLP recombinase system (Life Technologies).

We constructed pCS2934 (Additional file 1: Figure S5), which encodes MS2-DsRed expression under the control of an inducible promoter. A DNA fragment encoding CMV-TetO2-MS2-DsRed was obtained by digesting pCS2892 [22] with BglII and AvrII, and purifying the 1800 bp fragment by gel extraction. The backbone pCS2722 was digested with BglII and AvrII and the two DNA fragments were ligated together to form pCS2934, which includes an expression cassette encoding the expression of an OFF control device downstream of a cassette which encodes the expression of the MS2-DsRed fusion gene driven by the CMV-TetO2 promoter. A detailed list of all plasmids used in this work is contained in Additional file 1: Table S2.

For the construction of all OFF control devices, the miRNAs were cloned downstream of the spacer region using the AgeI and ClaI restriction sites. The ribozyme switches and controls were cloned upstream of the spacer region using the BstBI and XmaI restriction sites. All miRNA and ribozyme components were constructed by ordering two ultramer oligonucleotides (Integrated DNA Technologies), which were reverse complements of each other at their 3′ ends. The oligonucleotide pairs were subjected to five rounds of annealing and extension using Taq polymerase (New England Biolabs) in a thermocycler. The resulting DNA fragments were subsequently digested and cloned. The sequences of all miRNAs and ribozymes used in this study are presented in Additional file 1: Table S3.

### Mammalian cell culture

HEK293 Flp-In T-REx cells (Life Technologies) were maintained in DMEM supplemented with 10% FBS at 37°C in a 5% CO2-humidified incubator. Stable transfection of HEK293 Flp-In T-REx cell lines was performed using the Flp-In recombinase system (Life Technologies) according to the manufacturer’s instructions to generate isogenic stable cell lines. Stable integrants were selected using 200 μg/ml hygromycin B (Life Technologies), whereas stable cell lines were maintained in 100 μg/ml hygromycin B.

### Measuring fluorescent reporter expression through flow cytometry assays

miRNA induction experiments were performed by first seeding 24-well cell culture plates with stable cell lines at 2.5×10^5^ cells per well. After 48 hours, cells were trypsinized and subjected to flow cytometry analysis on a Cell Lab Quanta SC MPL (Beckman Coulter, Brea, CA), and the resulting data were analyzed using the FlowJo software (Tree Star, Ashland, OR). For experiments utilizing doxycycline induction, fresh media containing 0, 0.05, 0.2, 0.8, 3.2, or 50 ng/mL doxycycline was added to the wells 24 hours after seeding and measured by flow cytometry as described above. Cells were initially gated for viability by electronic volume and side scatter. GFP and DsRed fluorescence of viable cells were measured through 525- and 670-nm bandpass filters, respectively, after excitation with a 488-nm laser. The levels of the fluorescent reporters were calculated as the median fluorescence of the stable cell population (Additional file 1: Figure S6). All fluorescent reporter levels were normalized to the corresponding fluorescent reporter level from cells harboring a control construct lacking a miRNA under the same conditions. At least two biological replicates were included in each experiment, and reported data are the averages of at least three independent experiments. Error bars represent ±1 standard deviation.

### Measuring miRNA levels through Taqman assays

The levels of miRNAs in the cells were quantified using custom Taqman small RNA assays (Applied Biosystems, Carlsbad, CA) according to the manufacturer’s instructions. Cells at different experimental conditions were plated in duplicate in 96-well tissue culture plates at a concentration of 10,000 cells per well. After 48 hours, the cells in one well were trypsinized and counted in a hemocytometer to obtain an accurate count of the number of cells per well for each experimental condition. The cells in the remaining well were lysed by adding 50 μl of Taqman lysis buffer and incubating for 8 minutes. The lysis reaction was stopped with the addition of 5 μl of stop buffer, resulting in a total volume of 55 μl of lysate per sample. A fixed volume of 5 μl of this lysate was used as input into the reverse transcription (RT) reaction.

Input RNA was reverse transcribed using the TaqMan miRNA Reverse Transcription Kit and custom miRNA-specific stem-loop primers (Applied BioSystems) in a small-scale RT reaction (1.387 μl of H_2_O, 0.5 μl of 10X reverse transcription buffer, 0.063 μl of RNase inhibitor (20 units/μl), 0.05 μl of 100 mM dNTPs with dTTP, 0.33 μl of multiscribe reverse transcriptase, 1.67 μl of input RNA; components other than the input RNA were prepared as a larger volume master mix). The reaction was run on an iCycler iQ system (BioRAD, Hercules, CA) at 16°C for 30 min, 42°C for 30 min, and 85°C for 5 min. The RNU 6B Taqman miRNA control was used to normalize the C_t_ values of the experimental miRNAs and to confirm the cell quantities measured by hemocytometer counting. Real-time PCR was carried out on an iCycler iQ thermocycler (BioRAD) at 95°C for 10 minutes, followed by 40 cycles of 95°C for 15 seconds and 60°C for 1 minute. The C_t_ was calculated using available RT-PCR Miner software [41], which automatically detects C_t_ and efficiency values for each PCR reaction. The mean of the resulting C_t_ values for the target gene of each sample were subtracted from the mean C_t_ value for the control gene. The resulting values were then converted from log_2_ to linear scale and normalized to the value from the sample lacking any miRNA with the same concentration of ligand.

### Measuring binding properties between ribozyme switches and protein ligands

The binding properties between the protein-responsive ribozyme switches or RNA aptamers and the corresponding protein ligands (i.e., MS2, E2F1) were determined according to previously described methods [24]. Briefly, ribozyme switches, aptamers, and appropriate controls were transcribed from DNA templates using the MEGAshortscript T7 kit (Life Technologies) and purification of the transcription products was performed using the RNA Clean & Concentrator Kit (Zymo Research) according to the manufacturers’ instructions. In all experiments, the HBS-N running buffer (GE Healthcare) (10 mM HEPES, 150 mM NaCl, pH 7.4) was used with no MgCl_2_ present, to prevent cleavage of the ribozyme. A dilution series of the target was prepared in running buffer using a minimum of five concentrations for kinetic analysis. For each sample concentration, the ribozyme switch was captured onto the sample flow cell (FC2) for 30 seconds at a flow rate of 10 μL/minute, the target solution was injected over both flow cells at a flow rate of 30 μL/minute to monitor target association, and running buffer was injected over both flow cells at a flow rate of 30 μL/minute to monitor target dissociation.

Data processing and analysis were performed using Biacore X100 Evaluation Software version 2.0 (GE Healthcare, San Francisco, CA). A double-referencing method was performed to process all datasets [42,43]. Data from the sample flow cell (FC2) were referenced first by subtracting data from the reference flow cell (FC1) to correct for bulk refractive index changes, nonspecific binding, injection noise, matrix effects, and baseline drift. The reference-subtracted data (FC2-FC1) were double-referenced with a blank injection of running buffer to account for any systematic drift over the course of the injection. The double-referenced data were fit to a 1:1 binding model for kinetic analysis to calculate the *k*_*a*_ and *k*_*d*_ between the ribozyme and the protein. The overall dissociation constant, *K*_D_, was calculated from *k*_*a*_*, and k*_*d*_.

### Gel-based ribozyme cleavage assays

Gel-based ribozyme cleavage assays were performed as previously described [23]. Briefly, all gel-based ribozyme cleavage assays were performed in a physiologically relevant reaction buffer (50 μl) composed of 500 μM MgCl_2_, 100 mM NaCl, and 50 mM Tris–HCl (pH 7.5) at 37°C. A 12-nt RNA blocking sequence that is complementary to part of the ribozyme transcript was inserted at the 5′ end of the sTRSV HHRz transcript to generate a cis-blocked ribozyme construct. In the reaction volume, 75 nM of full-length RNA was first incubated with 2.5 μM DNA activator strand (5′-AAACAACTTTGTTTGTTTCCCCC-3′), which is ordered from Integrated DNA Technologies as an oligonucleotide with standard desalting, for 2 min to activate the blocked RNA. A zero time-point aliquot was taken before initiating the self-cleavage reaction with the addition of MgCl_2_ and the indicated concentration of input protein. Reactions were quenched after 20 min with addition of 3 volumes of RNA stop/load buffer (95% formamide, 30 mM EDTA, 0.25% bromophenol blue, 0.25% xylene cyanol) and placed on ice. Samples were heated to 95°C for 5 min, snap cooled on ice for 5 min, and size-fractionated on a denaturing (8.3 M urea) 10% polyacrylamide gel at 25 W for 45–60 min. Gels were exposed overnight on a phosphor screen and imaged on a FX Molecular Imager (BioRAD). The relative levels of the full-length transcript and cleaved products were determined by phosphorimaging analysis.

## Additional file

Additional file 1:
**Text S1.** Model description. **Figure S1.** Overview of ribozyme switch design. **Figure S2.** Non-cleaving ribozyme controls for different spacer lengths. **Figure S3**. Schematic representation of model. **Figure S4.** Characterization of E2F1-A1 riboswitch. **Figure S5.** Base plasmids used to create all plasmids stably integrated into HEK293 Flp-In cells in this work. **Figure S6**. Representative flow cytometry plots for HEK293 Flp-In cells stably expressing OFF control device constructs. **Table S1.** Description of parameters used in the model. **Table S2.** List of plasmids used in this work. **Table S3.** Sequences of ribozymes and miRNAs used in this work.

## References

[1] Lienert F, Lohmueller JJ, Garg A, Silver PA (2014). Synthetic biology in mammalian cells: next generation research tools and therapeutics. Nat Rev Mol Cell Biol.

[2] Jaenisch R, Young R (2008). Stem cells, the molecular circuitry of pluripotency and nuclear reprogramming. Cell.

[3] Hahn WC, Weinberg RA (2002). Modelling the molecular circuitry of cancer. Nat Rev Cancer.

[4] Slusarczyk AL, Lin A, Weiss R (2012). Foundations for the design and implementation of synthetic genetic circuits. Nat Rev Genet.

[5] Win MN, Liang JC, Smolke CD (2009). Frameworks for programming biological function through RNA parts and devices. Chem Biol.

[6] Bartel DP (2009). MicroRNAs: target recognition and regulatory functions. Cell.

[7] Shukla GC, Singh J, Barik S (2011). MicroRNAs: Processing, Maturation, Target Recognition and Regulatory Functions. Mol Cell Pharmacol.

[8] Lewis BP, Burge CB, Bartel DP (2005). Conserved seed pairing, often flanked by adenosines, indicates that thousands of human genes are microRNA targets. Cell.

[9] Culler SJ, Hoff KG, Smolke CD (2010). Reprogramming cellular behavior with RNA controllers responsive to endogenous proteins. Science.

[10] Tigges M, Marquez-Lago TT, Stelling J, Fussenegger M (2009). A tunable synthetic mammalian oscillator. Nature.

[11] Deans TL, Cantor CR, Collins JJ (2007). A tunable genetic switch based on RNAi and repressor proteins for regulating gene expression in mammalian cells. Cell.

[12] Leisner M, Bleris L, Lohmueller J, Xie Z, Benenson Y (2010). Rationally designed logic integration of regulatory signals in mammalian cells. Nat Nanotechnol.

[13] Beisel CL, Chen YY, Culler SJ, Hoff KG, Smolke CD (2011). Design of small molecule-responsive microRNAs based on structural requirements for Drosha processing. Nucleic Acids Res.

[14] Beisel CL, Bayer TS, Hoff KG, Smolke CD (2008). Model-guided design of ligand-regulated RNAi for programmable control of gene expression. Mol Syst Biol.

[15] An CI, Trinh VB, Yokobayashi Y (2006). Artificial control of gene expression in mammalian cells by modulating RNA interference through aptamer-small molecule interaction. RNA.

[16] Bloom RJ, Winkler SM, Smolke CD (2014). A quantitative framework for the forward design of synthetic miRNA circuits. Nat Methods.

[17] Yi TM, Huang Y, Simon MI, Doyle J (2000). Robust perfect adaptation in bacterial chemotaxis through integral feedback control. Proc Natl Acad Sci U S A.

[18] Ang J, McMillen DR (2013). Physical constraints on biological integral control design for homeostasis and sensory adaptation. Biophys J.

[19] Ballarino M, Pagano F, Girardi E, Morlando M, Cacchiarelli D, Marchioni M (2009). Coupled RNA processing and transcription of intergenic primary microRNAs. Mol Cell Biol.

[20] Win MN, Smolke CD (2007). A modular and extensible RNA-based gene-regulatory platform for engineering cellular function. Proc Natl Acad Sci U S A.

[21] Wei KY, Chen YY, Smolke CD (2013). A yeast-based rapid prototype platform for gene control elements in mammalian cells. Biotechnol Bioeng.

[22] Bloom RJ, WInkler SM, Smolke CD. A quantitative framework for the forward design of synthetic miRNA circuits. Nat Methods. 2014; in press. 10.1038/nmeth.3100 25218181

[23] Kennedy AB, Liang JC, Smolke CD (2013). A versatile cis-blocking and trans-activation strategy for ribozyme characterization. Nucleic Acids Res.

[24] Chang AL, McKeague M, Liang JC, Smolke CD (2014). Kinetic and equilibrium binding characterization of aptamers to small molecules using a label-free, sensitive, and scalable platform. Anal Chem.

[25] Liang JC, Chang AL, Kennedy AB, Smolke CD (2012). A high-throughput, quantitative cell-based screen for efficient tailoring of RNA device activity. Nucleic Acids Res.

[26] Mukherji S, Ebert MS, Zheng GX, Tsang JS, Sharp PA, van Oudenaarden A (2011). MicroRNAs can generate thresholds in target gene expression. Nat Genet.

[27] Kim DH, Saetrom P, Snove O, Rossi JJ (2008). MicroRNA-directed transcriptional gene silencing in mammalian cells. Proc Natl Acad Sci U S A.

[28] Hirao I, Spingola M, Peabody D, Ellington AD (1998). The limits of specificity: an experimental analysis with RNA aptamers to MS2 coat protein variants. Mol Divers.

[29] Ishizaki J, Nevins JR, Sullenger BA (1996). Inhibition of cell proliferation by an RNA ligand that selectively blocks E2F function. Nat Med.

[30] Kennedy AB, Vowles JV, d’Espaux L, Smolke CD (2014). Protein-responsive ribozyme switches in eukaryotic cells. Nucleic Acids Res.

[31] Purnick PE, Weiss R (2009). The second wave of synthetic biology: from modules to systems. Nat Rev Mol Cell Biol.

[32] Shimoga V, White JT, Li Y, Sontag E, Bleris L (2013). Synthetic mammalian transgene negative autoregulation. Mol Syst Biol.

[33] Elowitz MB, Leibler S (2000). A synthetic oscillatory network of transcriptional regulators. Nature.

[34] Stapleton JA, Endo K, Fujita Y, Hayashi K, Takinoue M, Saito H (2012). Feedback control of protein expression in mammalian cells by tunable synthetic translational inhibition. ACS Synth Biol.

[35] Rosenfeld N, Young JW, Alon U, Swain PS, Elowitz MB (2007). Accurate prediction of gene feedback circuit behavior from component properties. Mol Syst Biol.

[36] Corish P, Tyler-Smith C (1999). Attenuation of green fluorescent protein half-life in mammalian cells. Protein Eng.

[37] pDsRed-Express-DR Vector Information [Brochure]. 2005. Mountain View, CA: Clontech Laboratories, Inc.

[38] Becskei A, Serrano L (2000). Engineering stability in gene networks by autoregulation. Nature.

[39] Gardner TS, Cantor CR, Collins JJ (2000). Construction of a genetic toggle switch in Escherichia coli. Nature.

[40] Kumar D, An CI, Yokobayashi Y (2009). Conditional RNA interference mediated by allosteric ribozyme. J Am Chem Soc.

[41] Zhao S, Fernald RD (2005). Comprehensive algorithm for quantitative real-time polymerase chain reaction. J Comput Biol.

[42] Katsamba PS, Park S, Laird-Offringa IA (2002). Kinetic studies of RNA-protein interactions using surface plasmon resonance. Methods.

[43] Myszka DG (1999). Improving biosensor analysis. J Mol Recognit.

