# Supplementary material for: Synthetic feedback control using an RNAi-based gene-regulatory device
Source: J Biol Eng. 2015 Apr 14;9:5. doi: 10.1186/s13036-015-0002-3 (PMC4403951; doi:10.1186/s13036-015-0002-3)
Supplement: Additional file 1: — Text S1. Model description. Figure S1. Overview of ribozyme switch design. Figure S2. Non-cleaving ribozyme controls for different spacer lengths. Figure S3. Schematic representation of model. Figure S4. Characterization of E2F1-A1 riboswitch. Figure S5. Base plasmids used to create all plasmids stably integrated into HEK293 Flp-In cells in this work. Figure S6. Representative flow cytometry plots for HEK293 Flp-In cells stably expressing OFF control device constructs. Table S1. Description of parameters used in the model. Table S2. List of plasmids used in this work. Table S3. Sequences of ribozymes and miRNAs used in this work. [file 13036_2015_2_MOESM1_ESM.pdf]

Synthetic feedback control using an RNAi-based control device

Bloom RJ, Winkler SM, and Smolke CD

Supplementary Information

Supplementary Text 1. Model description

Supplementary Figure 1. Overview of ribozyme switch design

Supplementary Figure 2. Non-cleaving ribozyme controls for different spacer lengths

Supplementary Figure 3. Schematic representation of model

Supplementary Figure 4. Characterization of E2F1-A1 riboswitch

Supplementary Figure 5. Base plasmids used to create all plasmids stably integrated into HEK293 Flp-In cells in this work

Supplementary Figure 6. Representative flow cytometry plots for HEK293 Flp-In cells stably expressing OFF control device constructs

Supplementary Table 1. Description of parameters used in the model

Supplementary Table 2. List of plasmids used in this work

Supplementary Table 3. Sequences of ribozymes and miRNAs used in this work

References

Supplementary Text 1. Description of mathematical model

Variable definitions:
G_I = nascent OFF control device transcript (without miRNA)
G_M = full-length OFF control device transcript
$G_{I\text{-}Bound}$ = nascent OFF control device transcript (without miRNA) bound to input ligand
$G_{M\text{-}Bound}$ = full-length OFF control device transcript bound to input ligand
miR = RISC-associated mature miRNA
P = ligand that binds the ribozyme switch
TG = target gene mRNA
TP = target gene protein
$k_{trs\text{-}OFF}$ = transcription rate of the OFF control device transcript
$k_{deg\text{-}OFF}$ = degradation rate of the full-length OFF control device transcript
k_{cleave} = cleavage rate of the unbound ribozyme switch
$k_{cleave\text{-}bound}$ = cleavage rate of the ligand-bound ribozyme switch

k_a = association rate constant of ligand and ribozyme switch
k_d = dissociation rate constant of ligand and ribozyme switch
k_{mature} = rate of transition from G_I to G_M
k_{miR} = rate constant describing formation of mature miRNA from primary miRNA
$k_{deg\text{-}miR}$ = degradation rate constant of mature miRNA
K_m = Concentration of G at which miR degradation of the transcript is half of the maximum
k_{deg} = degradation rate constant of target gene mRNA
k_{cat} = catalytic constant for miR degradation of target gene transcript

We developed a simple mathematical model describing the rate of microRNA formation resulting from our OFF control device. To accomplish this, we track the transcription of the OFF control device and divide it into two distinct steps. The first step is the transcription of the upstream ribozyme. The transcript in which the upstream ribozyme is transcribed and the downstream microRNA is not yet transcribed we call a nascent transcript, (G_I). A full-length transcript, (G_M), encodes the entire OFF control device, including the downstream microRNA(s), and is formed from a nascent transcript with a rate constant, k_{mature}.

Both full-length and nascent transcripts include a ribozyme switch that is able to bind a ligand of interest, (P), with an association rate constant, k_a, and a dissociation rate constant, k_d. We define the transcripts bound to the ligand as new species, $G_{I\text{-}Bound}$ and $G_{M\text{-}Bound}$. The ribozyme switch cleaves at a rate k_{cleave} when unbound and at the rate $k_{cleave\text{-}B}$ when bound to the ligand. The model assumes that after ribozyme cleavage the transcript is rapidly degraded and no further transcription or microRNA formation can occur.

Mature miRNAs are formed from the pri-miRNA in the full-length transcripts at a rate k_{miR}. We assume that once a pre-miRNA is formed from the pri-miRNA through microprocessor cleavage, the transcript that harbored the pri-miRNA is rapidly degraded.

These processes are described by the coupled ordinary differential equations provided below.

$$\frac{dG_I}{dt} = k_{trs-OFF} - k_{cleave}G_I - k_aPG_I + k_dG_{I-Bound} - k_{miR}G_M - k_{mature}G_I \tag{1}$$

$$\frac{dG_M}{dt} = k_{mature}G_I - k_{cleave}G_M - k_aPG_M + k_dG_{M-Bound} - k_{process}G_M - k_{deg-OFF}G_M \tag{2}$$

$$\frac{dG_{I-Bound}}{dt} = k_aPG_{I-Bound} - k_dG_{I-Bound} - k_{cleave-bound}G_{I-Bound} - k_{mature}G_{I-Bound} \tag{3}$$

$$\frac{dG_{M-Bound}}{dt} = k_{mature}G_{I-Bound} - k_{cleave-bound}G_M + k_aPG_M - k_dG_{M-Bound} - k_{miR}G_{M-Bound} - k_{deg-OFF}G_{M-Bound} \tag{4}$$

$$\frac{dmiR}{dt} = k_{miR}(G_M + G_{M-Bound}) - k_{deg-miR}miR \tag{5}$$

The level of target gene expression that results from different levels of miRNA within the cell is calculated from a previously described model[1]. Briefly, we describe miRNA-mediated transcript degradation using a Michaelis-Menten like term in which the RISC-associated mature miRNA complex (*miR*) acts as an enzyme and the target gene mRNA (*TG*) acts as the substrate.

$$\frac{dTG}{dt} = k_{trs} - k_{deg}TG - k_{cat} * miR\frac{TG}{K_m + TG} \tag{6}$$

From this, the equation for target protein formation is:

$$\frac{dTP}{dt} = k_{tln} - k_{degP}TP \tag{7}$$

Both k_{cat} and K_m are fitted parameters and are unique to each miRNA-target gene pair. The parameters for both GFP- and DsRed-targeting miRNAs were determined previously[1] using least-squares non-linear regression curve fit for the implicit equation using Graphpad prism software and bounding the K_m with a maximum value of 10,000 nM, in accordance with values reported in the literature[2].

The series of differential equations used in the model (equations 1-7) was solved using an ordinary differential equation solver in MATLAB (Mathworks, Ipswich, MA).

Parameters that were not obtained experimentally were estimated using a non-linear least squares optimization method using the MATLAB Optimization toolbox. A summary of the model parameters and how they were derived is provided in Supplementary Table 1.

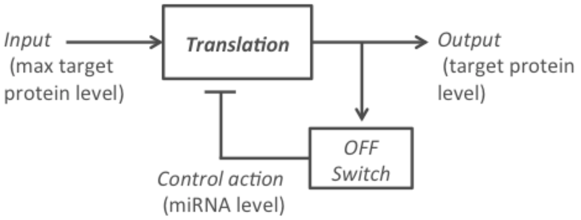

Using these parameters, we modified the model slightly to describe the case of the OFF control device used to exert negative feedback control. Rather than having two variables to represent both the ligand of interest, *P*, and the target protein, *TP*, in this case the target gene protein is the input ligand of interest, and thus these variables are represented by a single variable, *TP*. The feedback system can be represented using the block diagram above, where the maximum protein level is the input, target protein level is the output, and the miRNA level is the control action. The series of differential equations was solved in the same way as described for the OFF control device case.

$$\frac{dG_I}{dt} = k_{trs-OFF} - k_{cleave}G_I - k_a(TP)(G_I) + k_dG_{I-Bound} - k_{miR}G_M - k_{mature}G_I \tag{8}$$

$$\frac{dG_M}{dt} = k_{mature}G_I - k_{cleave}G_M - k_a(TP)(G_M) + k_dG_{M-Bound} - k_{process}G_M - k_{deg-OFF}G_M \tag{9}$$

$$\frac{dG_{I-Bound}}{dt} = k_a(TP)(G_I) - k_dG_{I-Bound} - k_{cleave-bound}G_{I-Bound} - k_{mature}G_{I-Bound} \tag{10}$$

$$\frac{dG_{M-Bound}}{dt} = k_{mature}G_{I-Bound} - k_{cleave-bound}G_M + k_a(TP)(G_M) - k_dG_{M-Bound} - k_{miR}G_{M-Bound} - k_{deg-OFF}G_{M-Bound} \tag{11}$$

$$\frac{dmiR}{dt} = k_{miR}(G_M + G_{M-Bound}) - k_{deg-miR}miR \tag{12}$$

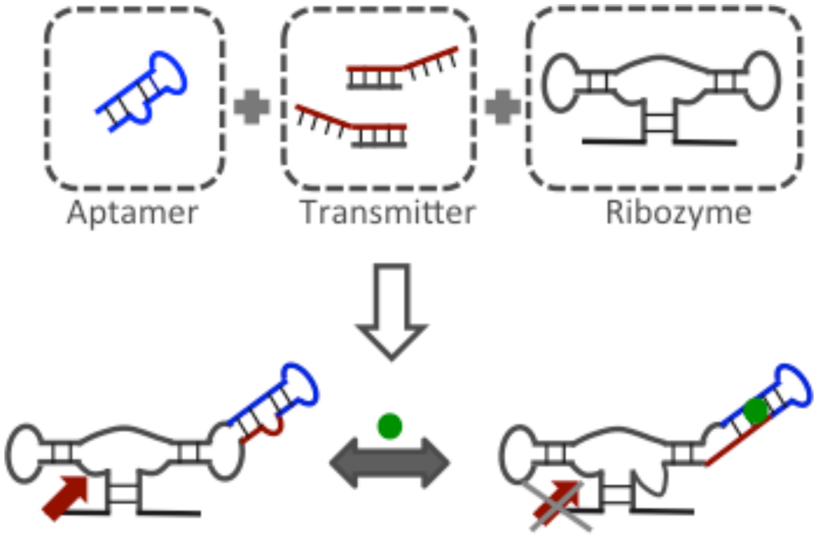

Supplemental Figure 1. Overview of ribozyme switch design. Ribozyme switches were designed by combining 3 distinct components: the sTRSV hammerhead ribozyme (grey), an aptamer (blue), and a transmitter (red) that joins the aptamer to loop II of the hammerhead ribozyme. With no ligand present, the ribozyme switch primarily adopts a conformation in which the ribozyme component is catalytically active and will cleave. The ligand will bind to a conformation in which the aptamer is formed, which shifts the conformational distribution to this ribozyme-inactive conformation such that cleavage is reduced. The sequences used in each ribozyme switch are listed in Supplementary Table 3 using the same color scheme to indicate the individual components.

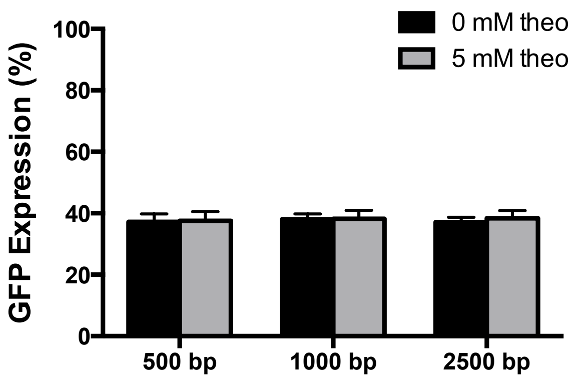

Supplemental Figure 2. Non-cleaving ribozyme controls for different spacer lengths. GFP expression for OFF control devices with a non-cleaving ribozyme placed at various distances upstream of a GFP-targeting miRNA.

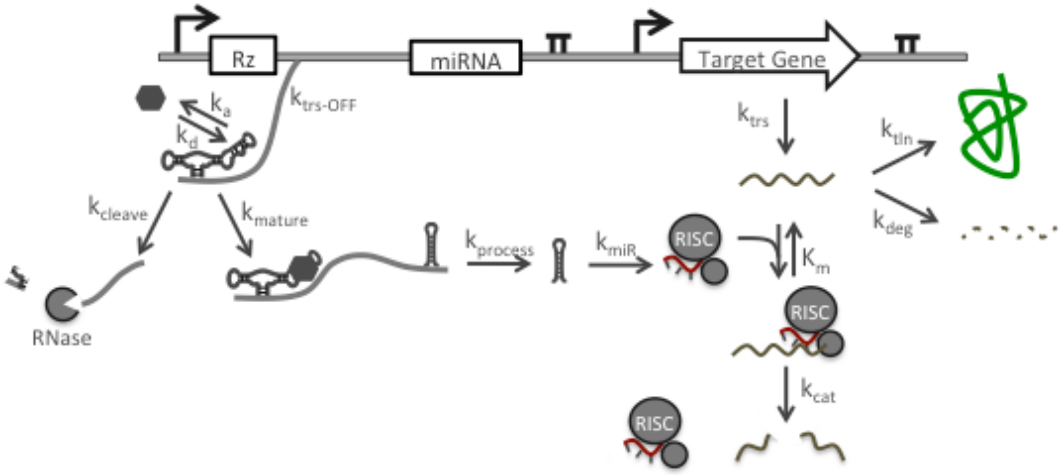

Supplementary Figure 3. **Schematic representation of the computational model.** Schematic representation of the rates and variables used in the series of differential equations representing the OFF control devices studied in this work.

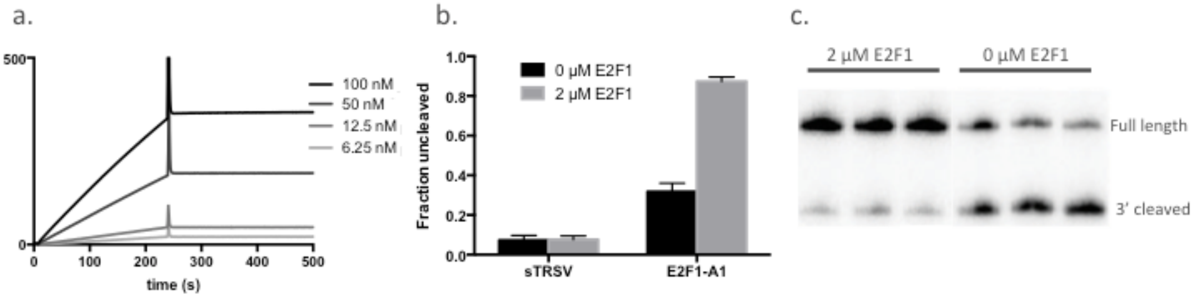

Supplementary Figure 4. Characterization of the E2F1-A1 ribozyme switch. (a) Representative sensorgrams associated with the surface plasmon resonance (SPR)-based *in vitro* binding assay for the E2F1-A1 ribozyme across a range of ligand concentrations. Binding and dissociation constants were determined as k_a: 3.80x10^4 M min^{-1}, k_d: 1.88x10^{-2} min^{-1}, K_D: 548.2 nM. b) Fraction of the ribozyme switch RNA uncleaved after a 20 min incubation in presence and absence of E2F1 protein. Phosphorimaging analysis of relative levels of the uncleaved, 5' cleaved, and 3' cleaved bands was used to determine the fraction cleaved. Error bars represent the standard deviation of two independent experiments. (c) Representative radiogel images from an end-point ribozyme cleavage assay for the E2F1-A1 ribozyme switch. Bands for the full-length uncleaved substrate and longer 3' cleaved product are shown, the shorter 5' cleaved product is omitted for clarity.

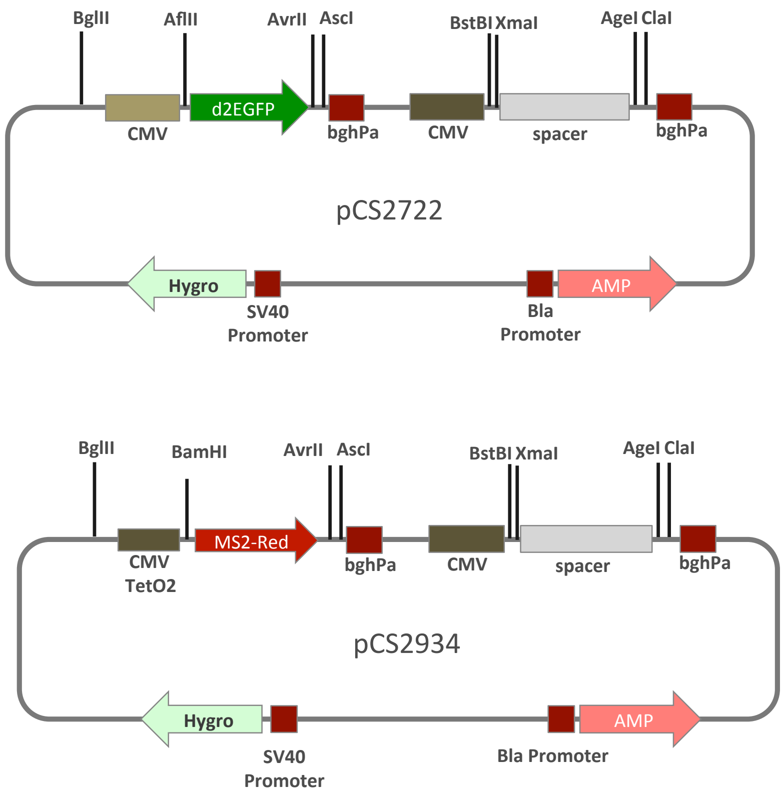

Supplementary Figure 5. Base plasmids used to create all plasmids stably integrated into HEK293 Flp-In cells in this work. pCS2722 and pCS2934 were used to create the vectors that were subsequently stably integrated into HEK293 Flp-In cells. Each of the plasmids contained a hygromycin resistance gene flanked by FRT recombination sites. miRNAs were cloned behind the spacer region using the AgeI and ClaI restriction sites and ribozymes were cloned between the BstBI and XmaI restriction sites.

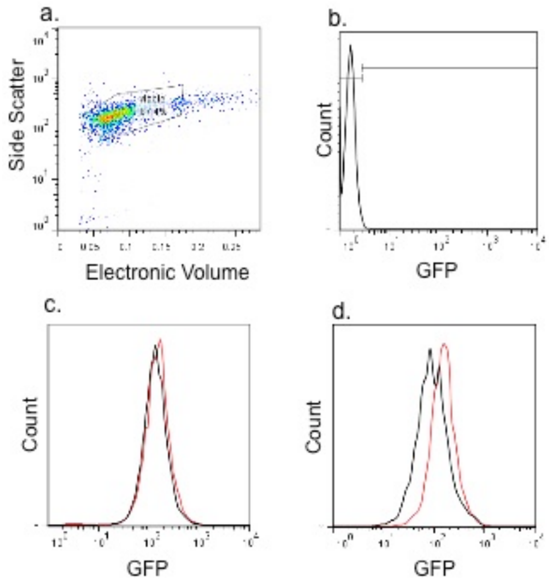

Supplementary Figure 6. Representative flow cytometry plots for HEK293 Flp-In cells stably expressing OFF control device constructs. (a) Plot showing side scatter vs. electronic volume for HEK293 cells, which was used to initially screen for viability. (b) A cell line lacking GFP was used to set the gate between the GFP negative population and GFP positive population. The GFP positive population was used in further data analysis. (c, d) Representative histograms depicting GFP expression from HEK293 Flp-In cell lines in the absence (c) and presence (d) of theophylline used in theophylline responsive control device experiments. Black: cell population expressing a theophylline OFF control device; red: cell population expressing no miRNA.

Supplementary Table 1. Description of parameters used in model

Parameter	value	Determination
$k_{trs\text{-}OFF}$	7.012 / min	fit to data with bounds of 1< $k_{trs\text{-}OFF}$<10
$k_{deg\text{-}OFF}$	0.434 /M-min	fit to data with bounds of .1< $k_{deg\text{-}OFF}$<10
$k_{transcribe}$	0.511 /min	fit to data
k_{miR}	0.198 /min	fit to data
$k_{deg\text{-}miR}$	0.014 /min	fit to data
MS2-A3 $k_{cleave\text{-}ribo}$	0.228 /min	calculated using gel-cleavage assay[3]
MS2-A3 $k_{cleave\text{-}bound}$	0.0563 /min	calculated using gel-cleavage assay[3]
L2B8-theo $k_{cleave\text{-}ribo}$	.140 /min	calculated using gel-cleavage assay[4]
L2B8-theo $k_{cleave\text{-}bound}$	.025/ min	calculated using gel-cleavage assay[4]
L2B8-theo k_a	9.0E6 /M-min	calculated using SPR-based assay[5]
L2B8-theo k_d	3.78 /min	calculated using SPR-based assay[5]
MS2-A3 k_a	4.52E4 /M-min	calculated using SPR-based assay[3]
MS2-A3 k_d	1.34E-2 /min	calculated using SPR-based assay[3]

Supplementary Table 2. List of plasmids used in this work

Cell Line	Expression Cassettes	Figure
Trex+pCS2407	P_{CMV}-d2EGFP-T_{bghpa} , P_{CMV}-strsv-cntrl - 500bp spacer -T_{bghpa}	1b, 1c, S1
Trex+pCS2873	P_{CMV}-d2EGFP-T_{bghpa} , P_{CMV}-strsv-cntrl - 1000bp spacer -T_{bghpa}	1b, 1c, S1
Trex+pCS2874	P_{CMV}-d2EGFP-T_{bghpa} , P_{CMV}-strsv-cntrl - 2500bp spacer -T_{bghpa}	1b, 1c, 4b, 4c, 4e, S1
Trex+pCS2875	P_{CMV}-d2EGFP-T_{bghpa} , P_{CMV}-strsv-cntrl - 500bp spacer - GFP-miR-T_{bghpa}	S1
Trex+pCS2876	P_{CMV}-d2EGFP-T_{bghpa} , P_{CMV}-strsv-cntrl - 1000bp spacer - GFP-miR-T_{bghpa}	S1
Trex+pCS2877	P_{CMV}-d2EGFP-T_{bghpa} , P_{CMV}-strsv-cntrl - 2500bp spacer - GFP-miR-T_{bghpa}	1b, 1c, S1
Trex+pCS2878	P_{CMV}-d2EGFP-T_{bghpa} , P_{CMV}-L2B8-A1 - 500bp spacer - GFP-miR-T_{bghpa}	1b, 1c
Trex+pCS2879	P_{CMV}-d2EGFP-T_{bghpa} , P_{CMV}-L2B8-A1 - 1000bp spacer - GFP-miR-T_{bghpa}	1b, 1c
Trex+pCS2880	P_{CMV}-d2EGFP-T_{bghpa} , P_{CMV}-L2B8-A1 - 2500bp spacer - GFP-miR-T_{bghpa}	1b, 1c
Trex+pCS2881	P_{CMV}-d2EGFP-T_{bghpa} , P_{CMV}-MS2-D5 - 2500bp spacer - GFP-miR-T_{bghpa}	4b, 4e
Trex+pCS2882	P_{CMV}-d2EGFP-T_{bghpa} , P_{CMV}-E2F1-A1 - 2500bp spacer - GFP-miR-T_{bghpa}	4c, 4e
Trex+pCS2883	P_{CMV}-MS2-DsRed-T_{bghpa} , P_{TetO2}- MS2-C3 - 2500bp spacer - T_{bghpa}	5d, 5e
Trex+pCS2884	P_{CMV}-MS2-DsRed-T_{bghpa} , P_{TetO2}- MS2-C3 - 2500bp spacer - GFP-miR - T_{bghpa}	5d, 5e

Supplementary Table 3. Sequences of ribozymes and miRNAs used in this work
Green indicates miRNA targeting sequence. Blue represent the aptamer sequence. Red indicates the transmitter. Bold indicates restriction sites used for cloning.

Construct	Sequence
miR-GFP	**ACCGGTGCGATCGC**GAACGGGTCCTGATACCAGCGTGAGCGAG CACAAGCTGGAGTACAACTATAGTGAAGCCACAGATGTATAGTT GTACTCCAGCTTGTGCCCGCCTACGCCCTTGGCAGCAGGGCCCGT TT**TTAATTAAATCGAT**
miR-DsRed	**ACCGGTGCGATCGC**GCGCCTGGAGGCTTGCTGAAGGCTGTATGC TGTTGGAGTCCACGTAGTAGTAGGTTTTGGCCACTGACTGACCTA CTACTGTGGACTCCAACAGGACACAAGGCCTGTGACTAGCACTC ACATGGAACAAATGGCC CGCG**TTAATTAAATCGAT**
Non-targeting miRNA	**ACCGGTGCGATCGC**GAACGGGTCCTGATACCAGCGTGAGCGAG CACAAGCTATCAACATGAGGTAGTGAAGCCACAGATGTACCTCA TGTTGATAGCTTGTGCCCGCCTACGCCCTTGGCAGCAGGGCCCGT TT**TTAATTAAATCGAT**
L2B8 theo (ribozyme switch)	**CAATTG**GCTGTCACCGGATGTGCTTTCCGGTCTGATGAGTCCGTT GTCCATACCAGCATCGTCTTGATGCCCTTGGCAGGGACGGGACGG AGGACGAAACAG**CCCGGG**
MS2-A3 (ribozyme switch)	**CAATTG**GCTGTCACCGGATGTGCTTTCCGGTCTGATGAGTCCGTG GTCCACCATCAGGGGACGGGACGGAGGACGAAACAGC**CCCGGG**
E2F1-A1 (ribozyme switch)	**CAATTG**GCGCTGTCACCGGAATCAAGGTCCGGTCTGATGAGTCC GTGGTCCTGTGATAAGTAGGACGGAGGTGGGGCCGGGACGGAGG ACGAAACAGCG**CCCCGGG**
miRNA only	**CAATTG**GCTGTCACCGGATGTGCTTTCCGGTCTGATGAGTCC GTGAGGACAAAACAGC**CCCGGG**

References

1 Bloom, R. J., WInkler, S. M., Smolke, C. D. A quantitative framework for the forward design of synthetic miRNA circuits. *Nature methods* **in press** (2014).

2 Haley, B. & Zamore, P. D. Kinetic analysis of the RNAi enzyme complex. *Nat Struct Mol Biol* **11**, 599-606, doi:10.1038/nsmb780 (2004).

3 Kennedy, A. B. e. a. Protein responsive ribozyme switches in eukaryotic cells. **in review**.

4 Kennedy, A. B., Liang, J. C. & Smolke, C. D. A versatile cis-blocking and trans-activation strategy for ribozyme characterization. *Nucleic Acids Res* **41**, e41, doi:10.1093/nar/gks1036 (2013).

5 Chang, A. L., McKeague, M., Liang, J. C. & Smolke, C. D. Kinetic and equilibrium binding characterization of aptamers to small molecules using a label-free, sensitive, and scalable platform. *Anal Chem* **86**, 3273-3278, doi:10.1021/ac5001527 (2014).
